# The association of the neighbourhood built environment with objectively measured physical activity in older adults with and without lower limb osteoarthritis

**DOI:** 10.1186/s12889-016-3347-8

**Published:** 2016-08-03

**Authors:** Erik J. Timmermans, Laura A. Schaap, Marjolein Visser, Hidde P. van der Ploeg, Alfred J. Wagtendonk, Suzan van der Pas, Dorly J. H. Deeg

**Affiliations:** 1 Department of Epidemiology and Biostatistics, EMGO Institute for Health and Care Research, VU University Medical Centre, De Boelelaan 1089A, 1081HV Amsterdam, The Netherlands; 2 Department of Health Sciences, Faculty of Earth and Life Sciences, VU University Amsterdam, De Boelelaan 1085, 1081HV Amsterdam, The Netherlands; 3 Department of Nutrition and Dietetics, Internal Medicine, VU University Medical Centre, De Boelelaan 1117, 1081BT Amsterdam, The Netherlands; 4 Department of Public and Occupational Health, EMGO Institute for Health and Care Research, VU University Medical Centre, Van der Boechorststraat 7, 1081 BT Amsterdam, The Netherlands; 5 Department of Environmental Economics, Institute for Environmental Studies, VU University Amsterdam, De Boelelaan 1085, 1081HV Amsterdam, The Netherlands

**Keywords:** Accelerometry, Geographic Information Systems, Neighbourhood built environment, Older population, Osteoarthritis, Physical activity

## Abstract

**Background:**

This study examined the associations of objectively measured neighbourhood built environment characteristics with objectively measured physical activity (PA) in older people with and without lower limb osteoarthritis (LLOA), and assessed whether these relationships differ between both groups.

**Methods:**

Data from the Dutch component of the European Project on OSteoArthritis were used. American College of Rheumatology classification criteria were used to diagnose LLOA (knee and/or hip osteoarthritis). Daily average time spent on total PA and separate PA intensity categories, including light PA, low-light PA, high-light PA, and moderate to vigorous PA, were measured using Actigraph GT3X accelerometers. Geographic Information Systems were used to measure street connectivity (number of street connections per km^2^) and distances (in km) to resources (health care resources, retail resources, meeting places, and public transport) within neighbourhoods. Multiple Linear Regression Analyses were used to examine the associations between measures of the neighbourhood built environment and PA, adjusted for several confounders.

**Results:**

Of all 247 participants (66–85 years), 41 (16.6 %) had LLOA. The time spent on any PA did not differ significantly between participants with and without LLOA (LLOA: Mean = 268.3, SD = 83.3 versus non-LLOA: Mean = 275.8, SD = 81.2; *p* = 0.59). In the full sample, no measures of the neighbourhood built environment were statistically significantly associated with total PA. Larger distances to specific health care resources (general practice and physiotherapist) and retail resources (supermarket) were associated with more time spent on PA in older people with LLOA than in those without LLOA. In particular, the associations of light and high-light PA with distances to these specific resources were stronger in participants with LLOA compared to their counterparts without LLOA.

**Conclusions:**

Specific attributes of the neighbourhood built environment are more strongly associated with PA in older people with LLOA than in those without LLOA. Knowledge on the relationship between objectively measured neighbourhood characteristics and PA in older people with and without LLOA could be used to inform policymakers and city planners about adaptation of neighbourhoods and their infrastructures to appropriately facilitate PA in healthy and functionally impaired older adults.

## Background

The majority of people with osteoarthritis (OA) of the lower limbs (hips and/or knees) do not engage sufficiently in physical activity (PA) [1]. Physical activity helps older people with lower limb osteoarthritis (LLOA) to reduce pain and improve functioning [2]. Furthermore, a physical active lifestyle reduces the risk of developing non-communicable diseases, such as cardiovascular disease, diabetes and several forms of cancer [3, 4].

The built environment encompasses a person’s surroundings which are human-made or modified, including physical structures and infrastructure [5]. The neighbourhood built environment is receiving growing attention in literature as a potential determinant of PA of older people [6]. Research on the influence of the neighbourhood built environment on PA in older people with disabilities is, however, limited [7]. For older people with LLOA, the built environment might be especially relevant as their pain and functional limitations might cause difficulties in overcoming environmental barriers towards PA [8]. To encourage older people with LLOA to be physically active, it is important to obtain knowledge on the association of PA with neighbourhood characteristics in this specific group.

To date, most studies on the association between PA and the neighbourhood built environment are conducted in the United States of America (USA) and Australia, and less is known about this relationship in Western European countries [9, 10]. Furthermore, most studies are based on self-reported PA and perceived assessment of the built environment [11]. Knowledge on the relationship between objectively measured neighbourhood characteristics and PA in older people is needed to appropriately facilitate PA of these persons.

The environmental docility hypothesis suggests that the behavior of a person is restricted or enhanced by environmental characteristics, depending on the functional capacity of the individual. It suggests that the less competent the individual, the greater the impact of environmental factors on that individual [12, 13]. Lower limb osteoarthritis is associated with joint pain, disability and functional limitations [14], which can be interpreted as a decrease of competence [15]. Older people with LLOA may experience more difficulties to overcome environmental barriers and may be more sensitive to PA facilitating factors in their neighbourhood environment compared to those without LLOA [8, 15].

Various perceived and objective characteristics of the neighbourhood built environment have been identified as facilitators and/or barriers for PA in older people [6]. Previous studies have shown that the presence of resources in the neighbourhood and a shorter perceived and objective distance to these resources are positively associated with PA of residents [16]. Furthermore, it has been shown that street connectivity, that is the number of street intersections per square kilometer within a neighbourhood, is positively associated with PA [17]. It has been suggested that a higher rate of interconnecting streets within a neighbourhood provides more travel route options and facilitates travelling through more direct routes, which in turn supports PA of residents [18].

Only a few studies have examined associations of neighbourhood built environment characteristics and PA in older persons with LLOA. White et al. [19] found that older people with knee OA whose neighbourhoods did not have parks and walking areas less frequently engaged in a regular fitness program and in social activities. A quantitative study by Keysor et al. [20] showed that people with symptomatic knee OA encountering community mobility barriers, such as lack of walking areas and public transportation, are more limited in their daily activities but do not perform these daily activities less frequently.

This study extends previous research by examining associations of objectively measured attributes of the neighbourhood built environment with objectively measured PA in Dutch older people. It is hypothesized that a higher rate of street connections in the neighbourhood is associated with more time spent on PA. Furthermore, it is expected that shorter distances to resources in the neighbourhood are associated with increased PA. It is hypothesized that these neighbourhood characteristics are more strongly related to PA in older people with LLOA than in those without the condition, because persons with LLOA might be more sensitive to attributes of the neighbourhood built environment.

## Methods

### Design and study sample

The study sample comprised men and women who participated in the Dutch component of the European Project on OSteoArthritis (EPOSA) and who originally participated in the Longitudinal Aging Study Amsterdam (LASA). The EPOSA-project and LASA have been described in detail previously [21, 22]. The EPOSA study focuses on the personal and societal burden of OA and its determinants in older persons in six European countries [21]. The LASA is an ongoing cohort study in the Netherlands that studies determinants, trajectories and consequences of physical, cognitive, emotional and social functioning in older persons [22]. In 2010, a random sample of 698 LASA-participants, aged 65–85 years, were invited by letter to participate in the EPOSA project of which 574 (82.2 %) agreed. A follow-up assessment after 12–18 months, during which 483 (84.1 %) persons participated. Reasons for non-response were death (*n* = 8), refusal (*n* = 79), and too ill health or frailty (*n* = 4).

Between January and November 2012, a random sample of 332 participants from 483 eligible participants at the EPOSA follow-up were asked to wear an accelerometer and to complete a daily activity diary for an 8-day period. In total, accelerometry data as well as data from the daily activity diary were obtained from 297 (89.5 %) participants. Participants with at least 4 valid days (≥10 h of wear time per day) of accelerometry data were included in the data analyses [23]. The analyses for this study are based on 247 older persons having data available on the presence of LLOA, objectively measured PA, the daily activity diary, measures on the neighbourhood built environment and the covariates.

The proportion of persons with a partner was higher in the included group (*n* = 247) compared to the excluded group (*n* = 85). Persons in the included group had a lower Body Mass Index (BMI) than those in the excluded group. There were no differences in other characteristics between the included and excluded group (data not shown).

### Measures

#### Objective measurement of physical activity

The Actigraph triaxial accelerometer (Model GT3X; ActiGraph, Pensacola, FL, USA) was used to objectively measure the time spent on PA. The accelerometer together with an instruction brochure which included pictures of how to properly wear the accelerometer were sent to the participants by mail. The accelerometer was attached to a 3 cm wide, tight elastic belt and was worn around the waist above the left iliac crest. Participants were briefed to wear the accelerometer for 8 consecutive days during waking hours. Furthermore, the participants were instructed to only remove the monitor right before going to bed and during showering, bathing, swimming and other water activities.

The accelerometry data were processed using ActiLife Data Analysis software (version 6.10.4) (ActiGraph, Pensacola, FL, USA). Physical activity was collected using 1-s epochs and were aggregated to 60-s epoch for data reduction. Non-wear time was defined by an interval of at least 60 consecutive minutes of zero activity counts on the y-axis, with allowance for 1–2 min of counts between 0 and 100 on this axis [23]. Wear time was determined by subtracting non-wear time from 24 h. Physical activity was measured as the mean time spent on PA in minutes per day. Furthermore, PA was measured as the daily mean time spent in each separate PA intensity category. These PA intensity categories were based on the number of counts per minute on the y-axis and were defined as: light PA (100–2019 counts/minute), which was further subdivided into low-light PA (100–759 counts/minute; eg, light household, and very slow walking) and high-light PA (760–2019 counts/minute; eg, slow walking), and moderate to vigorous PA (MVPA) (≥2020 counts/minute; eg, brisk walking, cycling, and running) [23–26].

### Objective measurement of the neighbourhood environment

Geographic Information Systems software, ArcGIS 10.1 (ESRI Inc, Redlands, CA, USA), was used to allocate the centroid of 6-digits post code areas of participants residential addresses to a neighbourhood, as defined by Statistics Netherlands and The Netherlands’ Cadastre, Land Registry, and Mapping Agency [27–29]. A geo-dataset with point locations of street connections in 2013 was available from The Netherlands’ Cadastre, Land Registry, and Mapping Agency via the Dutch National SDI [30]. A geo-dataset on the mean road distances of all residents in a neighbourhood to the closest resources in 2012 was available from Statistics Netherlands [28, 29, 31, 32].

The following objective neighbourhood built environment characteristics were measured: street connectivity (number of street connections per square kilometer within the neighbourhood), and mean road distance in km between occupied addresses and the closest visiting address of a resource in a neighbourhood. The following resources were included: the closest health care resource (general practice, general practice center, pharmacy, hospital with an outside clinic, hospital without an outside clinic, and physiotherapist), the closest retail resource (supermarket (≥150 m^2^), grocery store (eg greengrocer and baker), and department store), the closest meeting place (pub, cafeteria, and restaurant), and the closest public transport stop (train station and important public transport transfer station). The distances to these resources were measured on paved roads that could be used by cars. One-way traffic and entry prohibitions were not taken into account in this measurement. Furthermore, foot paths and bicycle lanes were not considered in this measurement [29, 32]. In the Netherlands, most paved roads in neighbourhoods can also be used by other road users, such as cyclists and walkers. It is assumed that this measurement also represents the distance to the closest resources for persons who do not use a car.

### Potential confounders

Potential confounders were age, sex (0 = men, 1 = women), partner status (0 = having no partner, 1 = having a partner), educational level (0 = lower educated than secondary education, 1 = secondary education or a higher level), urbanization grade, Body Mass Index (BMI), number of chronic diseases, anxiety, depression, functional limitations, and wear time of accelerometer.

Level of urbanization was assessed based on population size and density (1 = rural, <300 persons/km^2^ or <5000 inhabitants; 2 = intermediate, 5000–30000 inhabitants; 3 = urban, >300 persons/km^2^ and >5000 inhabitants).

Body Mass Index was calculated as weight in kilograms divided by height in square meters. Weight was measured to the nearest 0.1 kg using a calibrated scale. Height was measured to the nearest 0.001 m using a stadiometer. Number of chronic diseases was measured through self-reported presence of the following chronic diseases or symptoms that lasted for at least 3 months or diseases for which the participant had been treated or monitored by a physician: chronic non-specific lung disease, cardiovascular diseases, peripheral artery diseases, stroke, diabetes, and cancer. The number of chronic diseases other than LLOA was categorized into 0, 1, or 2 or more chronic diseases.

Anxiety and depressive symptoms were examined by the Hospital Anxiety Depression Scales (HADS-A and HADS-D) [33]. Both scales ranged from 0 to 21 and a cut-off level of 8 or more was used for presence of anxiety and depression.

Functional limitations were assessed by the physical function subscale of the Western Ontario and McMaster Universities OA Index (WOMAC) [34]. For the WOMAC, missing values were imputed according to the user manual and subscale scores were normalized resulting in subscale scores ranging from 0 (no difficulties) to 100 (extreme difficulties).

Wear time of accelerometer was determined by subtracting non-wear time from 24 h (see “Objective measurement of physical activity”).

### Potential effect modifier

A potential effect modifier was clinical LLOA. Algorithms for clinical OA of the hip and knee were developed based on the American College of Rheumatology (ACR) classification criteria [35]. Lower limb OA was defined as present when the participants had clinical OA in hip and/or knee. An extensive description of the diagnosis of OA in knee and hip is described elsewhere [21].

### Statistical analyses

Characteristics of older people with and without LLOA are presented using descriptive statistics. Differences in means were tested using Independent Sample T-Tests for normally distributed variables. Differences in medians were tested using Mann Whitney U Tests for skewed continuous variables and differences in frequencies were tested using Pearson Chi-square tests for frequencies.

Multiple Linear Regression Analyses were used to examine the associations between each of the neighbourhood built environment characteristics with the time spent on total PA and separate PA intensity levels. First, LLOA was assessed for potential effect modification by examining interaction effects between LLOA and each of the neighbourhood built environment characteristics in fully adjusted models. The interaction effects were considered significant at a *p*-value below 0.10 [36]. If an interaction term was significant, analyses were stratified for LLOA and group-specific associations between PA and the neighbourhood built environment were presented. In case the interaction effect was not significant, a pooled analysis (also adjusted for LLOA) was performed. Second, all associations of the neighbourhood built environment characteristics with PA were examined in models constructed step by step. Model 1 examined the association of each individual built environment characteristic with PA adjusted for sex and age. Model 2 additionally adjusted for all other confounders. Model 3 examined the associations between PA and distances to specific resources additionally adjusted for street connectivity. Because of multiple testing, the *p*-value was set to 0.01 in all models. Statistical analyses were performed in IBM SPSS Statistics (version 20.0).

## Results

The mean age of all 247 participants was 74.8 (SD = 5.6) years with an age-range of 66–85 years. Of all participants, 124 (50.2 %) were female. Forty-one (16.6 %) persons fulfilled the ACR classification criteria for knee and/or hip OA.

The characteristics of the participants with and without LLOA are presented in Table 1. The proportions of women and persons living in intermediate urban areas were higher in the LLOA-group than in the non-LLOA group. In addition, the participants with LLOA had a higher BMI and had more chronic diseases compared to the participants without LLOA. Furthermore, the proportion of persons with functional limitations was higher in the LLOA-group than in the non-LLOA group.

**Table 1:** Characteristics of the study sample stratified for lower limb osteoarthritis^a^

	All participants (*n* = 247)		Participants with LLOA (*n* = 41)		Participants without LLOA (*n* = 206)		*p*-value^b^
	*n*		*n*		*n*		
Characteristics							
Age in years (Mean (SD))	247	74.8 (5.6)	41	74.6 (5.3)	206	74.8 (5.6)	0.86
Sex (female) (n (%))	247	124 (50.2)	41	29 (70.7)	206	95 (46.1)	<0.01
Partner status (yes) (n (%))	247	158 (64.0)	41	22 (53.7)	206	136 (66.0)	0.13
Education (≥ secondary education) (n (%))	247	195 (78.9)	41	29 (70.7)	206	166 (80.6)	0.16
Urbanization grade (n (%))	247		41		206		0.03
Rural		53 (21.5)		7 (17.1)		46 (22.3)	
Intermediate urban		18 (7.3)		7 (17.1)		11 (5.3)	
Urban		176 (71.3)		27 (65.8)		149 (72.4)	
Body Mass Index in kg/m^2^ (Mean (SD))	244	27.3 (4.3)	41	29.3 (5.6)	203	26.9 (3.9)	0.01
Number of chronic diseases (n (%))	247		41		206		<0.01
0		99 (40.1)		8 (19.5)		91 (44.2)	
1		87 (35.2)		16 (39.0)		71 (34.5)	
≥ 2		61 (24.7)		17 (41.5)		44 (21.4)	
Functional limitations (0–100) (median (IQR))	232	3.1 (0.0–18.8)	36	34.9 (19.1–48.5)	196	1.5 (0.0–9.4)	<0.001
Anxiety (n (%))	234	20 (8.5)	37	5 (13.5)	197	15 (7.6)	0.24
Depression (n (%))	234	21 (9.0)	37	5 (13.5)	197	16 (8.1)	0.29

^a^ Abbreviations: *SD* Standard deviation, *IQR* Interquartile range, *LLOA* Lower limb osteoarthritis, *n* Number

^b^ p-value of observed differences between groups with and without LLOA

### Physical activity

On average, the participants had 6.5 (SD = 1.3) valid accelerometry days (Table 2). The majority of the study sample (55.5 %) had seven or more valid accelerometry days. In the full sample, mean wear time in minutes per day was 852.7 (SD = 65.8). On average, all participants were physically active at least at a light intensity level during 274.5 (SD = 81.4) minutes per day. This was 32.0 % of total wear time. In the full sample, participants spent 30.2 % of total wear time on light PA (24.4 and 5.8 % on low-light PA and high-light PA respectively). On average, all participants spent 1.8 % of total wear time on MVPA. The time spent on any PA did not differ significantly between participants with and without LLOA. Furthermore, the time spent in the specific PA intensity categories did not differ significantly between both groups (Table 2).

**Table 2:** Objectively measured physical activity in participants with and without lower limb osteoarthritis^a-c^

	All participants (*n* = 247)	Participants with LLOA (*n* = 41)	Participants without LLOA (*n* = 206)	*p*-value ^d^
Accelerometry				
Number of valid days (mean (SD))	6.5 (1.3)	6.8 (1.2)	6.5 (1.3)	0.18
Wear time in minutes/day (mean (SD))	852.7 (65.8)	853.8 (50.8)	852.4 (68.5)	0.91
Physical activity				
Total PA in minutes/day (mean (SD))	274.5 (81.4)	268.3 (83.3)	275.8 (81.2)	0.59
Light PA in minutes/day (mean (SD))	258.6 (74.5)	259.0 (77.6)	258.5 (74.1)	0.97
Low-light PA in minutes/day (mean (SD))	208.4 (51.9)	212.8 (53.9)	207.6 (51.6)	0.56
High-light PA in minutes/day (mean (SD))	50.1 (33.3)	46.3 (34.6)	50.9 (33.1)	0.41
Moderate to vigorous PA in minutes/day (median (IQR))	8.6 (3.0–22.1)	4.7 (1.4–12.7)	9.9 (3.3–23.5)	0.09

^a^ Abbreviations: *SD* Standard deviation, *IQR* Interquartile range, *LLOA*: Lower limb osteoarthritis, *n* Number, *PA* Physical activity

^b^ Participants with at least 4 valid days (≥10 h of wear time per day) of accelerometry data were included in the data analyses

^c^ Light PA: 100–2019 counts/minute; Low-light PA: 100–759 counts/minute; High-light PA: 760–2019 counts/minute; Moderate to vigorous PA: ≥2020 counts/minute

^d^ p-value of observed differences between groups with and without LLOA

### Neighbourhood built environment

The 247 participants lived in 139 neighbourhoods across 11 municipalities in the Netherlands. The objectively measured attributes of the neighbourhood built environment did not differ significantly between participants with and without LLOA (Table 3).

**Table 3:** Characteristics of the neighbourhood built environment in the study sample stratified for lower limb osteoarthritis ^a^

	All participants (*n* = 247)	Participants with LLOA (*n* = 41)	Participants without LLOA (*n* = 206)	*p*-value ^b^
Street Connectivity				
Number of street connections per km^2^ (mean (SD))	155.1 (87.9)	161.9 (75.2)	153.8 (90.3)	0.12
Distance to health care resources in km				
General practice (median (IQR))	0.6 (0.5–1.0)	0.5 (0.4–0.9)	0.7 (0.5–1.1)	0.10
General practice center (median (IQR))	5.5 (2.3–9.8)	6.8 (2.1–13.1)	5.4 (2.4–9.4)	0.90
Pharmacy (median (IQR))	0.8 (0.5–1.2)	0.6 (0.5–1.0)	0.8 (0.5–1.3)	0.08
Hospital with an outside clinic (median (IQR))	3.9 (1.9–6.8)	2.6 (1.6–7.0)	4.1 (2.0–7.0)	0.50
Hospital without an outside clinic (median (IQR))	4.9 (2.2–9.8)	6.8 (1.8–14.5)	4.9 (2.3–9.4)	0.90
Physiotherapist (median (IQR))	0.5 (0.4–0.8)	0.5 (0.4–0.7)	0.5 (0.4–0.8)	0.83
Distance to retail resources in km				
Large supermarket (median (IQR))	0.6 (0.5–0.9)	0.6 (0.4–0.8)	0.6 (0.5–0.9)	0.75
Grocery store (median (IQR))	0.5 (0.4–0.8)	0.5 (0.3–0.7)	0.5 (0.4–0.8)	0.87
Department store (median (IQR))	1.7 (1.0–4.1)	1.4 (0.9–2.9)	1.9 (1.0–4.3)	0.50
Distance to meeting places in km				
Pub (median (IQR))	0.7 (0.5–1.4)	0.7 (0.5–1.2)	0.7 (0.5–1.4)	0.98
Cafeteria (median (IQR))	0.5 (0.4–0.7)	0.4 (0.4–0.7)	0.5 (0.4–0.8)	0.89
Restaurant (median (IQR))	0.6 (0.4–0.9)	0.6 (0.3–0.8)	0.6 (0.4–0.9)	0.81
Distance to public transport in km				
Train station (median (IQR))	3.2 (1.9–11.4)	2.9 (2.1–12.5)	3.2 (1.7–11.4)	0.99
Important transfer station (median (IQR))	12.5 (4.6–18.9)	12.3 (4.3–20.9)	12.5 (4.7–18.8)	0.64

^a^ Abbreviations: *SD* Standard deviation, *IQR* Interquartile range, *km* kilometer, *LLOA* Lower limb osteoarthritis, *n* Number

^b^ p-value of observed differences between groups with and without LLOA

### Physical activity and street connectivity

In the full sample, the total time spent on PA was not statistically significantly associated with the number of street connections per square kilometer within the neighbourhood (Table 4). After adjustment for all confounders (Table 4; Model 2), a trend for a positive association between low-light PA and street connectivity was found (B = 0.07; *p* = 0.05). The associations between the PA measures and street connectivity did not differ between participants with and without LLOA.

**Table 4:** Associations between measures of the neighbourhood built environment and objectively measured physical activity^a,b^

	Total PA in minutes/day	Light PA in minutes/day	Low-light PA in minutes/day	High-light PA in minutes/day	Moderate to vigorous PA in minutes/day
	B (SE)	B (SE)	B (SE)	B (SE)	B (SE)
Street connectivity					
*Number of street connections per km* ^*2*^					
Model 1	0.01 (0.05)	0.02 (0.05)	0.06 (0.04)	−0.04 (0.02)	−0.01 (0.02)
Model 2	0.08 (0.05)	0.07 (0.05)	0.07 (0.04)^¥^	0.01 (0.02)	0.01 (0.02)
Distance to health care resources in km					
*General practice*					
Model 1	9.44 (5.19)	8.84 (4.78)	2.29 (3.48)	**6.55 (2.14)**	0.61 (247)
Model 2	1.75 (4.92)^c^	2.64 (4.86)^c^	0.71 (3.40)	1.97 (2.15)^c^	−0.88 (1.51)
Model 3	5.04 (5.23)^c^	5.65 (4.95)^c^	3.37 (3.60)	2.27 (2.29)^c^	−0.62 (1.61)
*General practice center*					
Model 1	0.19 (0.90)	0.19 (0.83)	−0.40 (0.60)	0.59 (0.37)	−0.01 (0.25)
Model 2	0.49 (1.15)	0.59 (1.09)	0.31 (0.79)	0.29 (0.50)	−0.10 (0.35)
Model 3	0.76 (1.16)	0.83 (1.10)	0.53 (0.79)	0.30 (0.51)	−0.07 (0.35)
*Pharmacy*					
Model 1	7.04 (4.46)	6.56 (4.10)	0.99 (2.98)	**5.57 (1.83)**	0.48 (1.27)
Model 2	0.22 (4.27)^c^	0.73 (4.04)^c^	−1.30 (2.95)	2.03 (1.86)^c^	−0.51 (1.30)
Model 3	3.61 (4.67)^c^	3.78 (4.42)^c^	1.25 (3.22)	2.48 (2.05)^c^	−0.20 (1.44)
*Hospital with an outside clinic*					
Model 1	−0.25 (1.30)	−0.41 (1.20)	−1.49 (0.86)	1.08 (0.54)^¥^	0.16 (0.37)
Model 2	−0.66 (1.46)	0.38 (1.38)	−0.85 (1.00)	1.22 (0.63)	0.28 (0.44)
Model 3	1.00 (1.47)	0.67 (1.39)	−0.58 (1.01)	1.26 (0.64)	0.33 (0.45)
*Hospital without an outside clinic*					
Model 1	0.14 (0.87)	0.15 (0.80)	−0.45 (0.58)	0.59 (0.36)	−0.01 (0.25)
Model 2	0.47 (1.13)	0.59 (1.07)	0.23 (0.78)	0.35 (0.49)	−0.11 (0.34)
Model 3	0.70 (1.13)	0.79 (1.07)	0.43 (0.78)	0.36 (0.50)	−0.09 (0.35)
*Physiotherapist in km*					
Model 1	9.41 (6.50)	10.59 (5.97)	1.41 (4.34)	**9.19 (2.66)**	−1.18 (1.84)
Model 2	0.52 (6.27)	3.83 (5.93)	−0.25 (4.32)	4.10 (2.72)^c^	−3.32 (1.90)
Model 3	4.47 (6.68)	7.78 (6.31)	3.03 (4.59)	4.72 (2.91)^c^	−3.30 (2.04)
Distance to retail resources in km					
*Supermarket*					
Model 1	7.44 (4.83)	5.67 (4.45)	−0.22 (3.23)	**5.89 (1.99)**	1.77 (1.37)
Model 2	1.60 (4.66)^c^	0.73 (4.40)^c^	−1.60 (3.21)	2.35 (2.03)^c^	0.87 (1.42)
Model 3	5.74 (5.12)^c^	4.18 (4.85)^c^	1.24 (3.53)	2.91 (2.25)^c^	1.54 (1.57)
*Grocery store*					
Model 1	0.26 (3.25)	−1.48 (2.99)	−3.40 (2.15)	1.92 (1.35)	1.74 (0.91)
Model 2	−0.95 (3.26)	−2.28 (3.08)	−2.65 (2.24)	0.37 (1.42)	1.18 (0.98)^c^
Model 3	1.22 (3.54)	−0.65 (3.35)	−1.14 (2.43)	0.49 (1.55)	1.73 (1.06)^c^
*Department store*					
Model 1	−0.72 (1.24)	0.65 (1.14)	−1.05 (0.82)	0.41 (0.52)	−0.07 (0.35)
Model 2	1.54 (1.49)	1.73 (1.40)	1.27 (1.02)	0.47 (0.65)	−0.19 (0.45)
Model 3	1.51 (1.48)	1.71 (1.40)	1.24 (1.02)	0.47 (0.65)	−0.19 (0.46)
Distance to meeting places in km					
*Pub*					
Model 1	−1.60 (3.10)	−2.64 (2.85)	−3.68 (2.05)	1.04 (1.29)	1.05 (0.87)
Model 2	−2.00 (3.11)	−2.48 (2.94)	−2.62 (2.14)	0.15 (1.36)	0.48 (0.95)
Model 3	−0.08 (3.40)	−0.94 (3.22)	−1.16 (2.33)	0.22 (1.49)	0.86 (1.04)
*Cafeteria*					
Model 1	7.55 (5.74)	7.01 (5.28)	−0.37 (3.83)	**7.38 (2.35)**	0.55 (1.63)
Model 2	0.28 (5.46)	1.15 (5.16)	−2.03 (3.76)	3.17 (2.37)	−0.87 (1.66)
Model 3	5.22 (6.12)	5.71 (5.79)	1.59 (4.21)	4.12 (2.67)	−0.49 (1.88)
*Restaurant*					
Model 1	4.13 (7.70)	6.15 (7.08)	−3.50 (5.12)	**9.64 (3.15)**	−2.02 (2.17)
Model 2	−8.71 (7.57)	−3.75 (7.18)	−6.43 (5.22)	2.68 (3.31)	−4.96 (2.29)
Model 3	−6.08 (7.84)	−1.17 (7.43)	−4.11 (5.38)	2.94 (3.43)	−4.91 (2.38)
Distance to public transport in km					
*Distance to train station in km*					
Model 1	−0.32 (0.82)	−0.59 (0.76)	−1.10 (0.54)^¥^	0.51 (0.34)	0.28 (0.23)
Model 2	−0.64 (0.89)	−0.94 (0.84)	−1.31 (0.60)	0.37 (0.39)	0.30 (0.27)
Model 3	−0.65 (0.88)	−0.94 (0.83)	−1.31 (0.60)	0.37 (0.39)	0.30 (0.27)
*Distance to important transfer station*					
Model 1	0.95 (0.57)†	0.97 (0.53)	0.48 (0.38)	0.49 (0.24)^¥^	−0.02 (0.16)
Model 2	0.62 (0.59)	0.66 (0.56)	0.46 (0.41)	0.19 (0.26)^¥^	−0.04 (0.18)
Model 3	0.59 (0.59)	0.63 (0.56)	0.44 (0.41)	0.19 (0.26)^¥^	−0.04 (0.18)

^a^ Abbreviations: *B* unstandardized coefficient, *SE* standard error, *km* kilometer, *PA* Physical activity; in bold: *p* < 0.01; ¥: 0.01 ≥ *p* < 0.05

^b^ Model 1 (based on 247 participants): adjusted for age and sex (reference category: men)

Model 2 (based on 226 participants): additionally adjusted for partner status (reference category: no partner), educational level (reference category: lower educated than secondary education), urbanization grade (reference category: rural area), Body Mass Index, number of chronic diseases (reference category: no chronic diseases other than lower limb osteoarthritis (LLOA)), anxiety (reference category: not anxious), depression (reference category: not depressed), functional limitations, wear time of accelerometer, and LLOA (reference category: no LLOA)

Model 3 (based on 226 participants): this model included all confounders and additionally adjusted for street connectivity

^c^ There was a significant interaction effect of this neighbourhood built environment measure with LLOA on the PA outcome measure. Therefore, the association in this model was not additionally adjusted for LLOA

### Physical activity and distance to health care resources

**Fig. 1:**
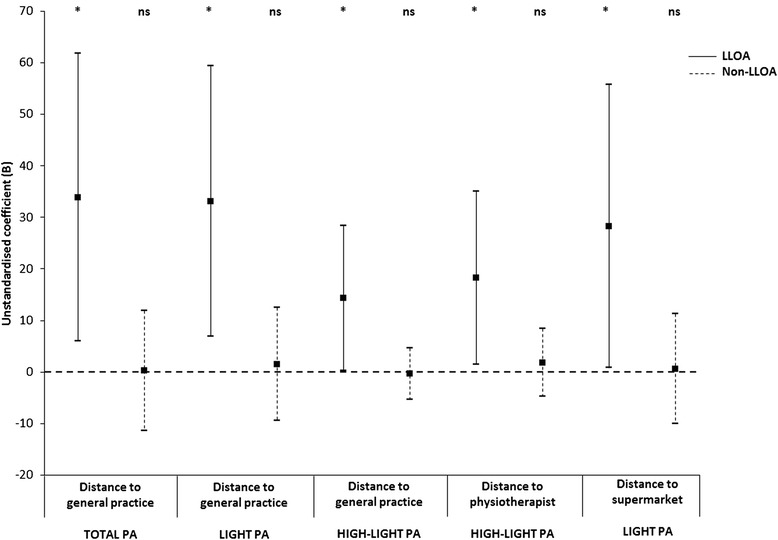
Associations between physical activity and distances to resources in older people with and without lower limb osteoarthritis^a-d^. ^a^ Abbreviations: LLOA: Lower limb osteoarthritis; PA: Physical activity. ^b^ Error bars represent 95 %-confidence intervals. Asterisk: *p* < 0.05; ns: not significant. ^c^ The associations are adjusted for age, sex (reference category: men), partner status (reference category: no partner), educational level (reference category: not better educated than secondary education), urbanization grade (reference category: rural area), Body Mass Index, number of chronic diseases (reference category: no chronic diseases other than lower limb osteoarthritis (LLOA)), anxiety (reference category: not anxious), depression (reference category: not depressed), functional limitations, wear time of accelerometer, and street connectivity. ^d^ Low-light PA: 100–759 counts/minute; High-light PA: 760–2019 counts/minute; Moderate to vigorous PA (MVPA): ≥2020 counts/minute

After adjustment for all confounders, including street connectivity, the distances to specific health care resources were not associated with total PA nor with any of the specific PA categories in the full sample (Table 4; Model 3). The associations of total PA, light PA, and high-light PA with distances to specific health care resources (general practice and physiotherapist) were stronger in participants with LLOA than in those without LLOA (Fig. 1).

### Physical activity and distance to retail resources

After adjustment for all confounders, including street connectivity, no statistically significant associations were found between the PA measures and the distances to specific retail resources in the full sample (Table 4; Model 3). The association of light PA with the distance to the closest supermarket was stronger in older people with LLOA (B = 28.30; *p* = 0.04) than in their counterparts without LLOA (B = 0.63; *p* = 0.91) (Fig. 1).

### Physical activity and distance to meeting places

After adjustment for all confounders, including street connectivity, there were no statistically significant associations between the PA measures and distances to specific meeting places in the full sample (Table 4; Model 3). The associations between the PA measures and distances to specific meeting places did not differ between participants with and without LLOA.

### Physical activity and distance to public transport

In the full sample, distances to public transport facilities were not significantly associated with any of the PA measures after adjustment for all confounders, including street connectivity (Table 4; Model 3). The associations between the PA measures and distances to public transport facilities did not differ between participants with and without LLOA.

## Discussion

This study examined whether objectively measured neighbourhood built environment characteristics are associated with PA in older people and assessed whether these relationships differed between those with and without LLOA. The results showed that distances to specific health care resources (general practice and physiotherapist) and retail resources (supermarket) were more strongly associated with time spent on PA in older people with LLOA than in those without LLOA. In particular, the associations of light and high-light PA with distances to these specific resources were stronger in older people with LLOA compared to their counterparts without LLOA.

As pain and disability become more severe, the capacity to adapt to the environment may decrease and environmental challenges, such as perceived and objectively larger distances to services, may become overwhelming. This may potentially lead to avoidance of challenging situations and restricted PA [12, 13, 37, 38]. Our results provide some supportive evidence for the environmental docility hypothesis and confirm that the less competent the individual, the greater the impact of environmental factors on that individual. The associations between some specific neighbourhood built environment characteristics were more strongly in older people with LLOA than in those without LLOA. However, in contrast to our expectations, the findings showed that objectively measured larger distances to specific resources were associated with more time spent on total PA, light PA and high-light PA by older people with LLOA than by those without LLOA. Individuals with LLOA may make more use of health care services than those without the condition and these facilities may be more important for older persons with LLOA than for their counterparts without LLOA [39]. As a consequence, older people with LLOA may be more motivated to participate in PA by larger distances to health care services compared to those without LLOA. Furthermore, the general practitioner might especially encourage older people with LLOA to be more physically active and, as a consequence, they just walk more to health care services and everyday retail resources, such as supermarkets. The association between the time spent on total PA and distances to specific health care services and retail opportunities may be stronger in older adults with LLOA than in those without LLOA, because it may be a greater effort for persons with LLOA to travel the distances to these specific resources and they may need more time to reach their destination. However, the findings do not show differences in PA between both groups.

This study also showed that a higher rate of interconnecting streets within a neighbourhood was marginally significantly associated with more time spent on low-light PA. This finding is in line with previous research [17]. It has been suggested that a higher rate of street connections within a neighbourhood provides more travel route options and facilitates direct travelling, which in turn supports being physically active [18]. Our finding implies that PA of older adults could be facilitated by increasing the density of street connections in the neighbourhood. In particular, PA of older persons could be improved by increasing the number of infrastructures that literally ask for PA, such as foot paths and bicycle lanes. Caution should be taken, however, when interpreting this result. Parks have a low road density, but do facilitate PA in older adults [16]. However, the presence of parks was not considered in the current study.

To our knowledge, this is the first population-based study that focused on the associations between objectively measured PA and neighbourhood built environment characteristics in older people with and without LLOA. An important strength of this study is the use of accelerometry to objectively assess time spent on PA, instead of self-reported PA measures. In contrast to previous studies which only focused on total PA [6], detailed analyses have been performed on specific PA intensity categories. Thereby, it was shown that specific characteristics of the neighbourhood built environment are, in particular, associated with time spent on light and high-light PA. Another strength is that the time spent on PA was collected for seven or more days for the majority of participants and therefore this study may truly reflect habitual behaviour of older people. Previous research on the relationship between objectively measured PA and built environment characteristics was mainly conducted in the USA and Australia [9, 10]. This study may contribute to a better understanding of PA-environment relationships in older adults from the general population in Western Europe.

Some limitations have to be acknowledged as well. In this study, the cut-off points of PA-categories were based on Troiano et al. [23] and Matthews et al. [24]. These cut-off points have been examined in adults. Although these cut-off points are widely used and generally accepted, they may not apply to older adults. There is evidence that the optimal cut-off points may vary for different age groups, due to dissimilar activity patterns, mechanical efficiency and the contrasting nature of movements at different life stages [40]. In this study, the geographical location of participants during their physical activities was not assessed, and hence information on context of PA was missing. The PA measures in this study thus also include PA that was performed at home or outside the neighbourhood. Another limitation was that the mean road distance in km of all residents in a neighbourhood to a specific resource was used as a proxy for the distance from the participants’ home to that specific resource. Furthermore, the number of persons with LLOA in this study is small and the variation in the neighbourhood built environment in the Netherlands is rather small. These methodological limitations might make it harder to gauge the true size of the associations between objectively measured neighbourhood built environment characteristics and PA. Finally, this study did not consider residential self-selection as a confounder in the relationship between the neighbourhood built environment characteristics and PA. Residential self-selection is the phenomenon that people choose where to live based on their needs and preferences [41]. It could be that people are more physically active because the neighbourhood built environment invites them to do so, but it could also be that people who like to be physically active tend to choose residential neighbourhoods conducive to exercising that preference.

In future research, specific cut-off points regarding PA categories for older adults have to be developed in order to accurately measure PA intensity in these individuals. Future research should not only focus on PA measures that are based on the number of counts per minute on the y-axis of an accelerometer, as more sophisticated methods (eg, pattern recognition) might become available in the near future. This may contribute to a more detailed measure of PA. Furthermore, future research on the associations between PA and characteristics of the neighbourhood built environment could make use of Global Position System (GPS) devices to make distinction in PA outside the neighbourhood, PA within the neighbourhood and PA at home. The use of GPS measures may provide more insight in the life space mobility area of older adults and helps to choose appropriate areas to study environmental influences on PA [42]. This study only focused on the associations of PA with objectively measured street connectivity and distances to specific resources. Future research is needed to obtain more insight in the associations of PA with other objective aspects of the neighbourhood built environment, such as safety, and presence and conditions of footpaths and bicycle lanes. In addition, persons do have perceptions of the built neighbourhood environment that may not equate with the objective measurements [43]. Future research is also needed to examine how perceived characteristics of the neighbourhood built environment are related to objective measurements and how these aspects are associated with PA. To obtain more insight into the relationship between perceived and objectively measured neighbourhood built environment characteristics and PA in healthy and functionally impaired older adults, future research could make use of ‘go-along’ interviews, alongside objective measures [44].

## Conclusions

In conclusion, this study shows that distances to specific neighbourhood resources are more strongly associated with PA in older people with LLOA than in those without LLOA. Knowledge on the relationship between objectively measured neighbourhood characteristics and PA in older people with and without LLOA could be used to inform policymakers and city planners about adaptation of neighbourhoods and their infrastructures to appropriately facilitate PA in healthy and functionally impaired older adults.

## Abbreviations

ACR, American College of Rheumatology; B, Unstandardized coefficient; BMI, Body Mass Index; CA, California; EPOSA, European Project on OSteoArthritis; FL, Florida; GPS, Global Position System; HADS-A, Hospital Anxiety Depression Scales-Anxiety Subscale; HADS-D, Hospital Anxiety Depression Scales-Depression Subscale; IBM SPSS Statistics, International Business Machines Corporation Statistical Package for the Social Sciences Statistics; IQR, Interquartile range; Km, Kilometer; LASA, Longitudinal Aging Study Amsterdam; LLOA, Lower limb osteoarthritis; MVPA, Moderate to vigorous physical activity; OA, Osteoarthritis; PA, Physical activity; SD, Standard deviation; SE, Standard error; USA, United States of America; WOMAC, Western Ontario and McMaster Universities Osteoarthritis Index.
